# p53-dependent Fas expression is critical for Ginsenoside Rh2 triggered caspase-8 activation in HeLa cells

**DOI:** 10.1007/s13238-014-0027-2

**Published:** 2014-03-13

**Authors:** Xiao-Xi Guo, Yang Li, Chao Sun, Dan Jiang, Ying-Jia Lin, Feng-Xie Jin, Seung-Ki Lee, Ying-Hua Jin

**Affiliations:** 1 Key Laboratory for Molecular Enzymology and Engineering of the Ministry of Education, College of Life Science, Jilin University, Changchun, 130012 China; 2 Faculty of Life Science and Technology, Kunming University of Science and Technology, Kunming, 650500 China; 3 College of Bio and Food Technology, Dalian Polytechnic University, Dalian, 116034 China; 4 College of Pharmacy and the Research Institute for Pharmaceutical Science, Seoul National University, Seoul, 157-742 Republic of Korea

**Keywords:** G-Rh2, Fas, p53, apoptosis

## Abstract

We have recently reported that Ginsenoside Rh2 (G-Rh2) induces the activation of two initiator caspases, caspase-8 and caspase-9 in human cancer cells. However, the molecular mechanism of its death-inducing function remains unclear. Here we show that G-Rh2 stimulated the activation of both caspase-8 and caspase-9 simultaneously in HeLa cells. Under G-Rh2 treatment, membrane death receptors Fas and TNFR1 are remarkably upregulated. However, the induced expression of Fas but not TNFR1 was contributed to the apoptosis process. Moreover, significant increases in Fas expression and caspase-8 activity temporally coincided with an increase in p53 expression in p53-non-mutated HeLa and SK-HEP-1 cells upon G-Rh2 treatment. In contrast, Fas expression and caspase-8 activity remained constant with G-Rh2 treatment in p53-mutated SW480 and PC-3 cells. In addition, siRNA-mediated knockdown of p53 diminished G-Rh2-induced Fas expression and caspase-8 activation. These results indicated that G-Rh2-triggered extrinsic apoptosis relies on p53-mediated Fas over-expression. In the intrinsic apoptotic pathway, G-Rh2 induced strong and immediate translocation of cytosolic BAK and BAX to the mitochondria, mitochondrial cytochrome c release, and subsequent caspase-9 activation both in HeLa and in SW480 cells. p53-mediated Fas expression and subsequent downstream caspase-8 activation as well as p53-independent caspase-9 activation all contribute to the activation of the downstream effector caspase-3/-7, leading to tumor cell death. Taken together, we suggest that G-Rh2 induces cancer cell apoptosis in a multi-path manner and is therefore a promising candidate for anti-tumor drug development.

**Electronic supplementary material:**

The online version of this article (doi:10.1007/s13238-014-0027-2) contains supplementary material, which is available to authorized users.

## Introduction

Apoptosis has been widely believed to play an important role in tissue development and homoeostasis maintenance. Dysregulation in the normal apoptotic process often leads to malignant transformation of cells (Favaloro et al., 2012; Brown and Attardi, 2005). In mammalian cells, apoptosis is initiated primarily by two pathways (Hengartner, 2000). The extrinsic apoptotic pathway involves the recruitment of death receptors, such as Fas and TNFR, by their specific ligands. The intrinsic apoptotic pathway depends on the depolarization of the mitochondrial outer membrane, which leads to the release of cytochrome c and the activation of caspase-9 (van Loo et al., 2002). Current strategies in cancer chemotherapy mainly focus on triggering or restoring these two apoptotic signaling cascades (Wong, 2011).

G-Rh2, a ginseng saponin in the protopanaxadiol family isolated from the root of ginseng C.A. has been shown to have an anticancer effect on a variety of tumor cells (Odashima et al., 1985; Kim et al., 2000; Jin et al., 2000; Ham et al., 2003; Oh et al., 2005; Ham et al., 2006; Xie et al., 2006; Kim et al., 2007). Our recent study has shown that both caspase-8 and caspase-9 were activated in G-Rh2 induced apoptosis of human hepatoma SK-HEP-1 cell (Guo et al., 2012). However, the exact molecular mechanisms of its pro-apoptotic functions remain poorly understood. In this study, we investigated two typical apoptotic pathways of G-Rh2-induced apoptosis in human cancer cell lines with different p53 mutation statuses. The data herein clearly showed that G-Rh2 triggers p53-dependent Fas expression and consequent activation of caspase-8 and p53-independent caspase-9-mediated intrinsic pathway to cause cancer cell death.

## Results

### G-Rh2 has an anti-proliferative effect on a variety of human cancer cells

**Figure 1:**
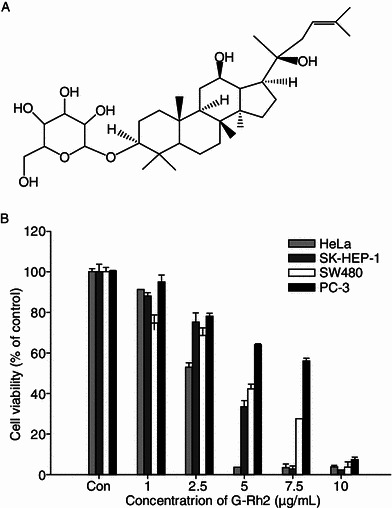
**Dose-dependent antiproliferative effects of G-Rh2 on human cancer cells**. (A) The chemical structure of G-Rh2. (B) HeLa, SK-HEP-1, SW480, and PC-3 cells were treated with G-Rh2 at different concentrations for 48 h. The cell viability was determined by MTT assay. The results represent three independent experiments in triplicate. The values from each treatment are expressed as a percentage relative to the control (100%). (mean ± SD)

The cytotoxic activity of G-Rh2 in the human tumor cell lines HeLa, SK-HEP-1, SW480, and PC-3 was assessed by MTT. Our previous study has shown that the expression of caspase-9 is significantly decreased in SK-HEP-1 and PC-3, and the expression of caspase-9 influences cell sensitivity to cytotoxic drugs, such as betulin or etoposide, which induces apoptosis via initiated caspase-9/-3 activation pathway by mitochondrial cytochrome c release (Li et al., 2010). Moreover, SW480 and PC-3 carry a mutant form of the p53 gene (Bamford et al., 2004; Nigro et al., 1989). The results (Fig. 1B, Table 1) showed that the cell viability of HeLa cells was remarkably inhibited by G-Rh2, with an IC_50_ value of 2.52 μg/mL, whereas SK-HEP-1 and SW480 cells were less sensitive to G-Rh2, with IC_50_ values of 3.15 μg/mL and 4.06 μg/mL, respectively. PC-3 cells were the least vulnerable to G-Rh2, with an IC_50_ value of 7.85 μg/mL, 3-fold higher than HeLa cells.

**Table 1:** The IC_50_ values for 48 h treatment of G-Rh2 in cell lines with different p53 mutation status and caspase-9 expression level

Cell line	TP53 status	Caspase-9 expression	IC_50_ (μg/mL)*
HeLa	WT	Normal	2.52 ± 0.05
SK-HEP-1	WT	Low	3.15 ± 0.03
SW480	MUT R273H	Normal	4.06 ± 0.02
PC-3	MUT A138del	Low	7.85 ± 0.03

*mean ± SD of three independent experiments

### Extrinsic and intrinsic pathways are simultaneously activated in G-Rh2-induced apoptosis

**Figure 2:**
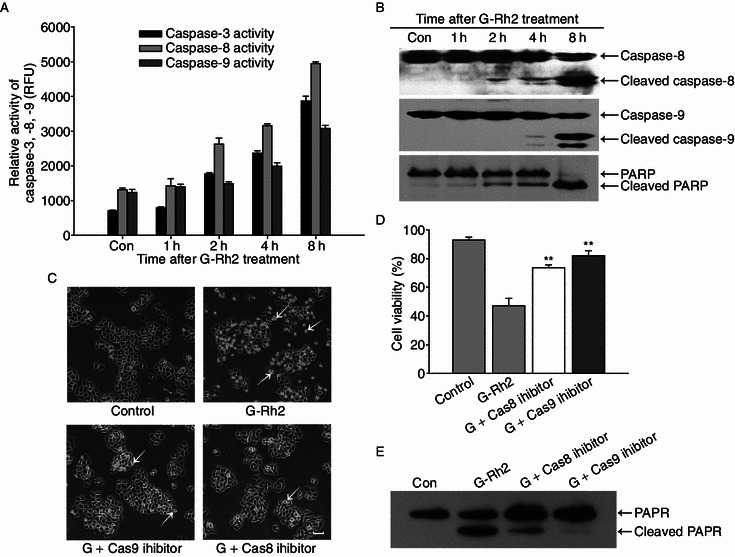
**Caspases-8, -9, and -3 were activated in G-Rh2-treated HeLa cells**. (A–B) HeLa cells were treated with 7.5 μg/mL G-Rh2 for indicated times. (A) The cell-free caspase-3, -8, and -9 activities were analyzed using specific substrates. Values are reported as the mean ± SEM of five experiments. (B) Equal amounts of cell extracts were analyzed by Western blotting. (C–E) HeLa cells were treated with G-Rh2 (25 μg/mL) or co-treated with G-Rh2 and caspase-8, or -9 inhibitor (100 μmol/L), in the presence of 2.5% serum for 8 h. (C) The cell morphology changes were photographed using microscopy (50×) Significant differences: **P < 0.01 compared with cells treated with G-Rh2 alone. The white arrows indicate blebbing cells undergoing apoptosis (bar, 50 μm). (D) The cell viability was determined by counting the blebbing and intact cells. The values from each treatment are expressed as an average proportion of intact cells in the total cell count (mean ± SD of three independent experiments). (E) The apoptotic status of cells was confirmed by Western blotting using specific antibodies against PARP and its cleaved form. Lane 1 represents cells treated with G-Rh2 alone, lane 2 represents cells co-treated with G-Rh2 and caspase-9 inhibitor, and lane 3 represents cells co-treated with G-Rh2 and caspase-8 inhibitor

To acquire more insight into the pro-apoptotic process of G-Rh2, we investigated the caspase-8, -9, and -3 activities in G-Rh2-treated HeLa cells in a time-dependent manner. We observed a notable increase in caspase-8 activity after 2 h G-Rh2 treatment, which occurred slightly earlier than that of caspase-9, which had an apparent rise 4 h after G-Rh2 treatment (Fig. 2A). The increase in caspase activity was also confirmed by Western blotting, which showed the cleavage of caspase-8 and -9 (Fig. 2B). To examine the significance of the extrinsic and intrinsic pathways in G-Rh2-induced apoptosis, we co-treated HeLa cells with G-Rh2 and specific peptide inhibitors for caspase-8 or caspase-9. Apoptosis progress was evaluated by determining cell morphology changes and caspase-3/-7 activation by PARP cleavage analysis. The data showed that cell apoptosis was remarkably attenuated by both caspase-8 and caspase-9 inhibitors compared with that in cells treated with G-Rh2 alone (Fig. 2C–E). All these results indicated that G-Rh2 might have the unique ability to simultaneously and independently activate both caspase-8 and caspase-9 in HeLa cells.

### Fas and TNF-R1 are up-regulated in G-Rh2-treated HeLa cells

**Figure 3:**
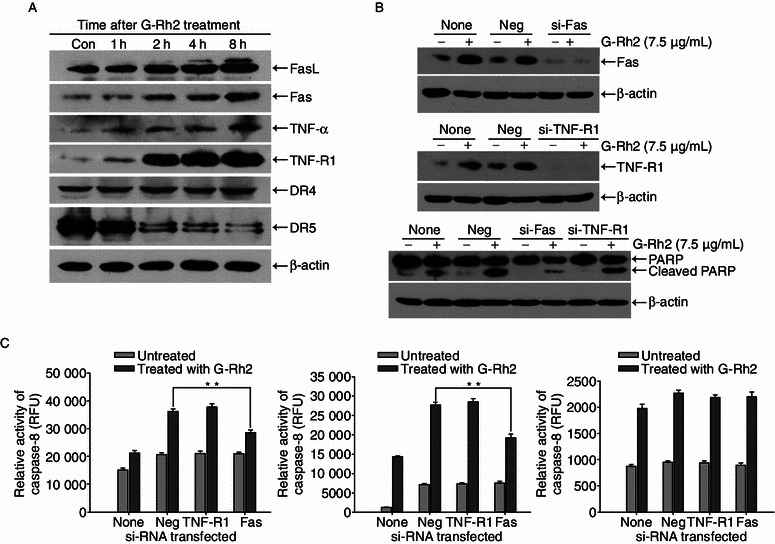
**G-Rh2 induced the expression of death receptors and the apoptosis induced by G-Rh2 in HeLa cells is dependent on Fas**. (A) HeLa cells were treated with 7.5 μg/mL G-Rh2 for indicated times. The cell lysates were analyzed by Western blotting. (B–C) HeLa cells were transfected with siRNA against Fas or TNF-R1 for 24 h before treatment with or without 7.5 μg/mL G-Rh2 for 6 h. Non-transfected cells and cells transfected with negative control RNA duplex served as controls. (B) Cell lysates were analysed by Western blotting. (C) The activity of caspase-8, -9, and -3 were determined as described in MATERIALS AND METHODS (Asterisks represent statistical significant differences with negative control, ** *P* < 0.01)

To further explore the process of the death receptor-initiated caspase-8 activation pathways, which was rarely investigated in previous studies. First, we detected the mRNA levels, by using RT-PCR, of five typical death receptors, Fas, TNF-R1, TNF-R2, DR4, and DR5, and three corresponding ligands, FasL, TNF-α, and TRAIL, in HeLa cells treated with 7.5 μg/mL G-Rh2 for 4 h. The results showed that the mRNA levels of Fas, TNF-α, TNF-R1, DR4, and DR5 were remarkably up-regulated after G-Rh2 treatment. No transcriptional changes were detected in FasL, TRAIL, and TNF-R2 (Fig. S1). We further examined the protein expression of Fas, FasL, TNF-α, TNF-R1, DR4, and DR5 in HeLa cells upon G-Rh2 treatment by Western blotting. The data showed that the expression of Fas, TNF-α, and TNF-R1 were up-regulated by G-Rh2 in a time-dependent manner, and the level of secreted FasL rose slightly. In addition, the expression of DR5 decreased but the expression of DR4 did not change with G-Rh2 treatment (Fig. 3A).

### Fas is the key factor for caspase-8 activation in G-Rh2-induced apoptosis

Because our data suggested that the pro-apoptotic function of G-Rh2 might largely depend on the up-regulation of Fas and TNF-R1, we examined the Fas or TNF-R1-mediated caspase-8 activation cascade by, respectively, silencing Fas or TNF-R1 via using small interfering RNAs against them in HeLa cells. After silencing either Fas or TNF-R1, the cells were treated with 7.5 μg/mL G-Rh2 for 6 h. The expression of Fas and TNF-R1 and PARP cleavage was determined by Western blotting and caspase-8, -9, and -3 activities were assayed. The results showed that the silencing of Fas significantly attenuated caspase-8 and caspase-3 activation and PARP cleavage, whereas silencing of TNF-R1 seemed to have no effect on G-Rh2-induced apoptosis. Meanwhile, caspase-9 activities were not influenced by either Fas or TNF-R1 silencing (Fig. 3B and 3C).

### G-Rh2-induced Fas expression is mediated by p53

**Figure 4:**
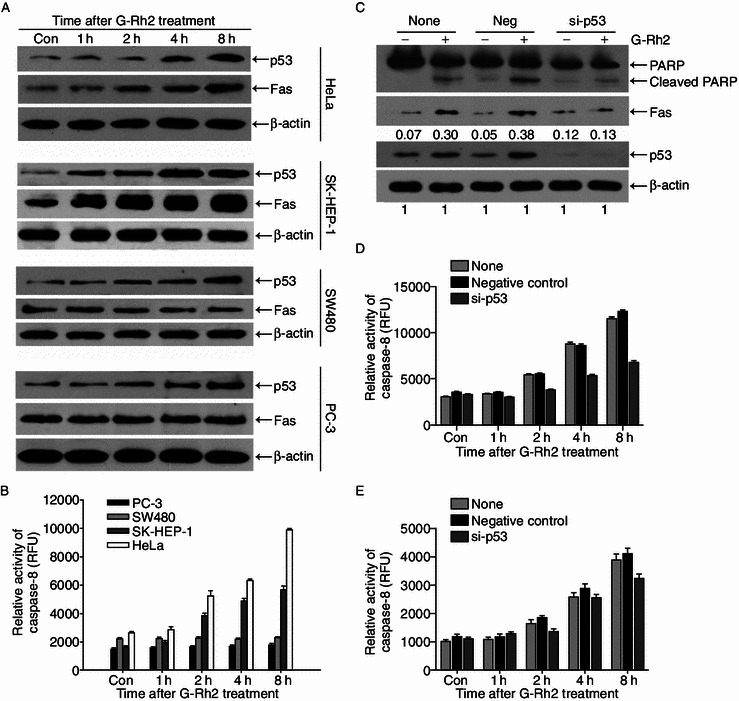
**G-Rh2 induces p53-dependent Fas expression in cancer cells**. (A) HeLa, SK-HEP-1, SW480, and PC-3 cells were treated with 7.5 μg/mL G-Rh2 for indicated times. The cell lysates were analyzed by Western blotting. (B) Caspase-8 activities of four cell lines were determined at indicated time points. (C) HeLa cells were transfected with a siRNA against p53 for 24 h before treatment with or without 7.5 μg/mL G-Rh2 for 6 h. Non-transfected cells and cells transfected with negative control RNA duplex served as controls. The cell lysates were analyzed by Western blotting. The numbers below the Fas bands represent each band’s relative abundance to the loading controls, which was quantified by Image-Pro Plus software. (D) HeLa cells were transfected with a siRNA against p53 for 24 h before treatment with or without 7.5 μg/mL G-Rh2 for 6 h and caspase-8 and -9 activities were determined as described in MATERIALS AND METHODS

Mutations in p53 genes occur in a large number of cancer cells (Bamford et al., 2004; Nigro et al., 1989; Zalcenstein et al., 2003), causing the resistance of cancer cells to anti-tumor reagents. A number of studies have been reported that the expression of the death receptor Fas (CD95) is controlled by wild-type p53 (Ham et al., 2003; Zalcenstein et al., 2003; Owen-Schaub et al., 1995; de la Monte et al., 1997; Takahashi, 1997; Müller et al., 1998; Embree-Ku et al., 2002; Schilling et al., 2009). Considering these facts and our experimental results, we hypothesized that Fas up-regulation after G-Rh2 treatment in human cancer cells might be related to p53 status. To examine this idea, we investigated Fas expression and caspase-8 activation upon G-Rh2 treatment in human cancer cells with wild type or mutated version of p53. The results showed that after treatment with G-Rh2, significant increases in Fas expression and caspase-8 activity coincided with an increase in p53 expression in p53-non-mutated HeLa and SK-HEP-1 cells. In contrast, Fas expression and caspase-8 activity remained constant with G-Rh2 treatment in p53-mutated SW480 and PC-3 cells (Fig. 4A and 4B). Moreover, p53 expression in these four cell lines increased in varying degrees, which suggests G-Rh2 may act as a stabilizer for p53 (Fig. 4A). The relationship between p53 and G-Rh2-induced Fas up-regulation in p53-silenced HeLa cells was also confirmed by Western blotting. As shown in Fig. 4C and 4D, G-Rh2-induced Fas up-regulation, caspase-8 activation, and PARP cleavage were remarkably attenuated in p53-silenced HeLa cells, compared with that of cells transfected with negative control siRNAs. Interestingly, the activation of caspase-9 seems to be not significantly influenced by p53 silencing (Fig. 4E)

### Mitochondrial-mediated caspase-9 activation in both p53 wild type HeLa cells and p53-mutated SW480 cells

**Figure 5:**
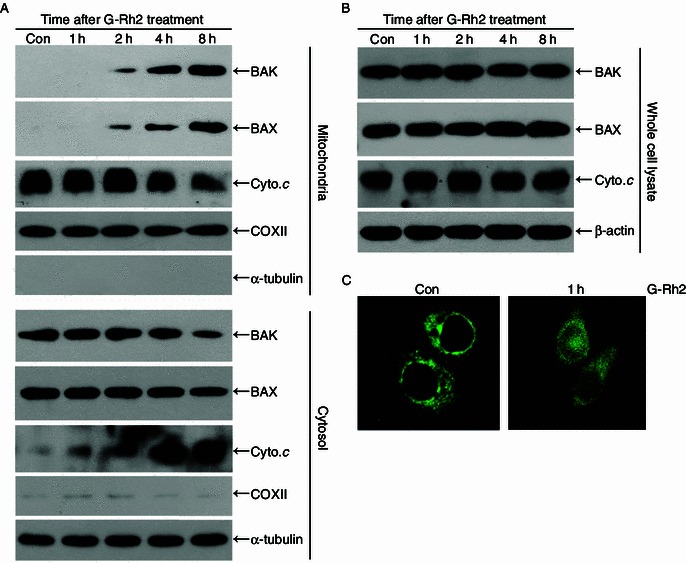
**G-Rh2 triggers the translocation of BAX and BAK, and cytochrome c release in HeLa cells**. HeLa cells were treated with 7.5 μg/mL G-Rh2 at indicated times. The cell fractions were isolated as described in MATERIALS AND METHODS. The (A) mitochondrial, and cytosolic fractions, and (B) total cell lysate were analyzed by Western blotting for BAK, BAX, cytochrome c, COX II, α-tubulin, and β-actin. (C) The cells were fixed and stained with anti-cytochrome c antibodies and analyzed by fluorescence microscope using appropriate filters for the visualization of green fluorescence resulting from the presence of FITC molecules

To gain more insight into the mechanism of G-Rh2-induced apoptosis, we examined the mitochondrial-mediated intrinsic pathway in HeLa cells. We treated HeLa cells with G-Rh2 for indicated times. Cell fractions were isolated and examined by Western blotting. The results demonstrated that the levels of mitochondrial BAK and BAX began to notably increase at 2 h after G-Rh2 treatment (Fig. 5A); at the same time, the levels of cytosolic cytochrome c increased in a similar time-dependent manner. In contrast, the levels of cytosolic BAK and BAX, and mitochondrial cytochrome c decreased in a time-dependent manner (Fig. 5A). However, the levels of total BAK and cytochrome c remained constant, whereas BAX was slightly upregulated during this process (Fig. 5B). Immunostaining analysis also showed that cytochrome c remarkably released from mitochondria upon G-Rh2 treatment (Fig 5C). These data implied that G-Rh2 effectively provoked the translocation of the pro-apoptotic proteins BAK and BAX from the cytosol to the mitochondria in HeLa cells, which in turn consequently induced cytochrome c release from the mitochondria to the cytosol (Fig. 5A and 5B), and therefore activated caspase-9 (Fig. 2A and 2B).

**Figure 6:**
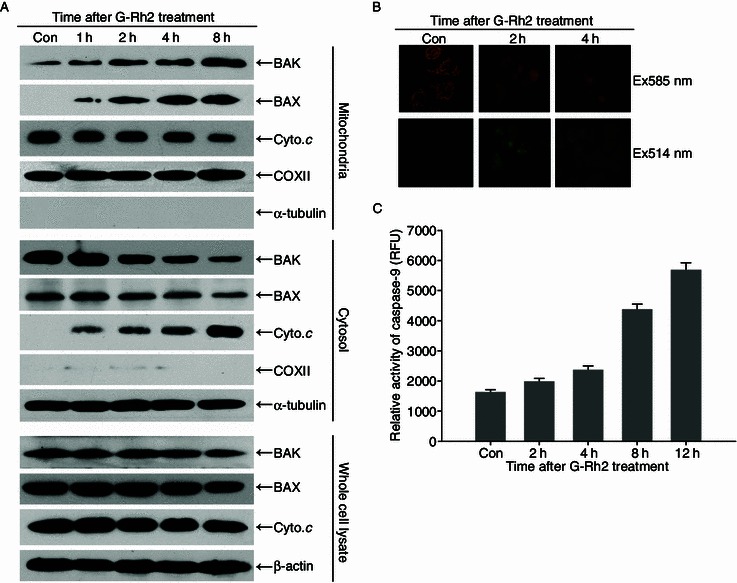
**G-Rh2 triggers the translocation of BAX and BAK, cytochrome c release, and consequently activation of caspase-9 in SW480 cells**. SW480 cells were treated with 7.5 μg/mL G-Rh2 at indicated times. The cell fractions were isolated as described in MATERIALS AND METHODS. (A) The mitochondrial and cytosolic fractions and total cell lysate were analyzed by Western blotting. (B) G-Rh2-induced dissipation of ΔΨm in SW480 cells. Red fluorescence represents the mitochondrial aggregate form of JC-1, indicating intact mitochondrial membrane potential. Green fluorescence represents the monomeric form of JC-1, indicating dissipation of ΔΨm. (C) At various time points, caspase-9 activity was assayed as described in MATERIALS AND METHODS. Values are reported as the mean ± SEM of five experiments

We also investigated the intrinsic apoptosis pathway in p53-mutated SW480 cells. The results demonstrated that the levels of mitochondrial BAK and BAX began to notably increase at 2 h after G-Rh2 treatment (Fig. 6A); at the same time, we detected, by JC-1 staining, the dissipation of mitochondrial membrane potential in G-Rh2 treated SW480 cells (Fig. 6B), which were coincident with the increase of cytosolic cytochrome c (Fig. 6A). Similar with HeLa cells, under the treatment of G-Rh2, the expression level of whole-cell BAK and cytochrome c remained constant. But different from the results of HeLa cells, the whole-cell BAX levels did not change, which may due to the loss of transcriptional function of mutated p53 in SW480 cells (Figs. 5 and 6A). These data implied that G-Rh2 effectively provoked the translocation of the pro-apoptotic proteins BAK and BAX from the cytosol to the mitochondria in SW480 cells, which in turn promoted the depolarization of mitochondria and consequently induced cytochrome c release from the mitochondria to the cytosol, and therefore activated caspase-9 (Fig. 6C), similar to that observed in G-Rh2-treated HeLa cells (Fig. 5). In contrast, caspase-8 was not activated during this process (Fig. 4B), indicating that, in SW480 cells, mutated p53 disables Fas upregulation and prevents the activation of caspase-8. These results implied that G-Rh2-induced cytochrome c release and caspase-9 activation is independent on p53-related and/or Fas-initiated caspase-8 activation.

## Discussion

For decades, anti-cancer compounds have been developed to target specific apoptosis-related proteins in certain signaling pathways. However, the expression of apoptosis-related genes varies widely in carcinoma cells, which gives cancer cells various sensitivities to anti-tumor drugs (Brown and Wilson, 2003; Galmarini and Galmarini, 2003; Liu, 2009). In this sense, multi-target drugs capable of triggering multiple apoptotic pathways will be ideal candidates for chemotherapy. Frequent down-regulation of the death receptor Fas (CD95) aids in apoptotic-escape of cancer cells, thereby promoting cancer progression and tumor evasion (Debatin and Krammer, 2004; Peter et al., 2005).

For the first time, we have shown that G-Rh2 induces apoptosis in human cancer cells by activating both extrinsic and intrinsic apoptosis pathways (Fig. 2). Activation of the extrinsic pathway by G-Rh2 significantly induced the over-expression of the death receptor Fas in a wild-type p53-dependent manner and thus, resulted in caspase-8 activation (Fig. 3). In agreement with our findings, a previous study observed that G-Rh2 induced Fas oligomerization in HeLa cells (Yi et al., 2009). Fas expression might reinforce the immune privilege of apoptosis-resistant tumor cells, which, in most cases, express low levels of Fas and thus, cannot sufficiently respond to apoptotic signal brought about by FasL in immune cells (Nagata, 1999; Strasser et al., 2009; Zhou et al., 1998). We also observed that p53 was up-regulated in targeted cells upon G-Rh2 treatment (Fig. 4A), which implies that G-Rh2 might increase the stability of p53 and prevent the ubiquitin-mediated degradation of p53 (Brooks and Gu, 2003).

Although another death receptor, TNF-R1, and its ligand, TNF-α, were also up-regulated after G-Rh2 treatment in HeLa cells, the increase in expression did not seem to contribute to G-Rh2-induced apoptosis (Fig. 3). TNFR1 is involved in death signalling; however, TNF-induced cell death only plays a minor role, compared to its overwhelming functions, in the inflammatory process. Its death-inducing capability is often masked by the anti-apoptotic effects of NF-κB (Gaur and Aggarwal, 2003). In any case, further investigation is warranted.

Activation of the intrinsic pathway mediated by G-Rh2 triggers caspase-9 activation by promoting strong and immediate translocation of both BAX and BAK from the cytosol to the mitochondria and, consequently, the release of cytochrome c from the mitochondria. Different from previous reports (Li et al., 2011; Kim and Jin, 2004), G-Rh2-induced mitochondrial pathway appeared not to be mediated by p53-dependent Bax overexpression in HeLa cells. The protein levels of Bax were not notably increased after G-Rh2 treatment (Fig. 5B) and the silencing of p53 did not attenuate the early stage activation of caspase-9 in G-Rh2 treated HeLa cells (Fig. 4E).

p53 genes are mutated in more than 50% of human cancer cell lines, which results in chemoresistance of those cell lines (Bamford et al., 2004; Kawamata et al., 2007; Andrews et al., 2004; Cappello et al., 2002). Importantly, G-Rh2 induced effective apoptotic cell death even in the p53-mutated cancer cell line SW480 via mitochondrial BAX and BAK translocation, which initiated the caspase-9 pathway (Fig. 6).

Taken together, the multi-path pro-apoptotic function and the unique capability of reinforcing the Fas/FasL pathway suggest that G-Rh2 may be a promising anti-tumor drug candidate. However, we found that G-Rh2 interacts with serum BSA (Data not shown) and its activity is attenuated by presence of serum (Li et al., 2011). Therefore, it is important to overcome the effect of serum in G-Rh2 based drug development. It will be also important to identify the precise cellular targets of G-Rh2, which may help to elucidate the mechanism of its anti-tumor functions and contribute to the development of more effective anticancer drugs.

## Materials And Methods

### Cell culture

HeLa, SK-HEP-1, SW480, and PC-3 cells were grown in Dulbecco’s Modified Eagle’s Medium (DMEM) supplemented with 10% heat-inactivated calf serum, 100 μg/mL of penicillin, and 100 μg/mL of streptomycin at 37°C in a humidified atmosphere containing 5% CO_2_.

### Determination of cell viability by MTT assay

Determination of cell viability was performed by using MTT assay (Sigma, St. Louis, MO), which was used to calculate the growth inhibition induced by increasing concentrations of drug. Briefly, exponentially growing cells were seeded into a 96-well plate at 1 × 10^4^ cells/well in triplicate. After incubation for 24 h, cells were treated with increasing concentration of G-Rh2 in serum free media for 48 h. At the end of treatment, 20 μL of MTT (5 mg/mL) was added to each well and incubated for an additional 4 h. The formazan grains formed by viable cells were solubilized with DMSO, and the color intensity was measured at 550 nm with an ELISA reader (BioTek Instruments, Winooski, VT).

### RNA purification and Reverse Transcription-PCR

Total RNA was isolated from HeLa cells with Tri Reagent (Molecular Research Centre, Inc. Cincinnati, OH) according to the manufacturer’s instructions. RNA was reverse-transcribed with an RNA PCR Kit (AMV, Takara Biotechnology, Dalian, China) and the supplied oligo dT primer using a thermal program of 42°C for 30 min, 99°C for 5 min, and 5°C for 5 min. Transcribed poly (A) RNA of pSPTet3 plasmid was used as a positive control RNA. PCR reactions were performed with Taq polymerase (Takara Biotechnology, Dalian, China) using the primers shown in Table S1. Supplied primers to β-actin were used to determine the mRNA level of β-actin, which served as an internal reference. PCR products were resolved on 1.5% agarose gels and visualized with ethidium bromide under ultraviolet light.

### Preparation of total protein in cell lysates

G-Rh2-treated cells in serum free media were washed with PBS, and cell pellets were re-suspended in lysis buffer containing 20 mmol/L Tris, pH 7.5, 0.5% Triton X-100, 2 mmol/L MgCl_2_, 1 mmol/L DTT, 1 mmol/L EGTA, 50 mmol/L β-glycerol phosphate, 25 mmol/L NaF, 1 mmol/L Na_3_VO_4_, 2 mg/mL leupeptin, 2 mg/mL pepstatin A, 2 mg/mL antipain, and 1 mmol/L PMSF for 1 h. The lysates were centrifuged at 12000 rpm for 15 min, and the supernatants were recovered for further protein analysis.

### Caspase activity assay

HeLa, SK-HEP-1, SW480, and PC-3 cells were treated with G-Rh2 (7.5 μg/mL) in serum free media for indicated time periods and then were harvested. Fifty micrograms of cell lysates were incubated with 200 nmol/L Ac-DEVD-AFC (for caspase-3), Ac-IETD-AFC (for caspase-8), and Ac-LEHD-AFC (for caspase-9) in a reaction buffer containing 20 mmol/L HEPES, pH 7.4, 100 mmol/L NaCl, 10 mmol/L DTT, 0.1% CHAPS, and 10% sucrose at 37°C for 1 h. The reaction was monitored by fluorescence emission at 535 nm and excitation at 405 nm.

### JC-1 staining and ΔΨm measurement

Loss of mitochondrial membrane potential (ΔΨm) was assessed by fluorescence microscope (IX71, Olympus) by JC-1 (Beyotime, China) staining. SW480 cells were stained with JC-1 for 20 min at 37°C after incubation with G-Rh2 (7.5 μg/mL) in serum free media for indicated times. The fluorescence was detected with a microplate reader (Tecan infinite 200). The wavelengths of excitation and emission were 514 nm and 529 nm respectively for detection of monomeric form of JC-1. 585 nm and 590 nm were used to detect aggregation of JC-1. Red emission of the dye represented a potential-dependent aggregation in the mitochondria, reflecting ΔΨm. Green fluorescence represented the monomeric form of JC-1, appearing in the cytosol after mitochondrial membrane depolarization.

### Preparation of mitochondrial and cytosolic extracts

Preparation of mitochondrial and cytosolic extracts was performed using the Mitochondria Isolation Kit (Pierce Biotechnology, Rockford, IL) for cultured cells according to the manufacturer’s instructions. Isolated mitochondria were solubilized in lysis buffer containing 20 mmol/L Tris, pH 7.5, 0.5% Triton X-100, 2 mmol/L MgCl_2_, 1 mmol/L DTT, 1 mmol/L EGTA, 50 mmol/L β-glycerol phosphate, 25 mmol/L NaF, 1 mmol/L Na_3_VO_4_, 2 mg/mL leupeptin, 2 mg/mL pepstatin A, 2 mg/mL antipain, and 1 mmol/L PMSF for 1 h. The lysate was centrifuged at 12000 rpm for 15 min, and the supernatant containing the mitochondrial extract was collected.

### Western blotting analysis

Antibodies against cytochrome c, poly (ADP-ribose) polymerase (PARP), and β-actin were purchased from Santa Cruz Biotechnology. Antibodies against caspase-8, caspase-9, Fas, TNFR1, TNF-α, DR4, DR5, and p53 were purchased from Abcam (Hong Kong). Horseradish peroxidase (HRP)-conjugated secondary antibodies were purchased from Pierce Biotechnology. Equal amounts of cell lysates were separated by SDS-PAGE and electrotransferred onto immobilon transfer membranes (Millipore, Bedford, MA). The membranes were blocked with 5% skim milk and probed with the indicated antibodies. The blots were washed and incubated with an HRP-coupled anti-mouse or anti-rabbit IgG antibody, followed by detection with ECL-enhanced chemiluminescence detection reagents (Amersham Biosciences, Piscataway, NJ). β-actin, α-tubulin or COXII were used as loading controls.

### RNA interference

siRNA duplexes for Fas, TNF-R1, p53, and non-targeting negative control siRNA were purchased from Bioneer. The siRNA sequences are listed in Table S2. 3 × 10^5^ HeLa cells in six-well dishes were transfected with a final concentration of 75 nmol/L siRNA duplexes using HiPerFect Transfection Reagent (Qiagen, Valencia, CA) according to the manufacturer’s instructions. After incubation for 24 h, the cells were treated with G-Rh2 (7.5 μg/mL) in serum free media for 6 h. Finally, the total protein in the cell lysates was prepared for caspase activity assay and Western blotting analysis of Fas, TNF-R1, p53, β-actin, and PARP.

### Immunostaining analysis

The cells were cultured overnight on sterile glass cover slips in 24-well plates. The cells were treated with G-Rh2 (7.5 μg/mL) for 2 h to induce apoptosis and subsequently fixed with 95% ethanol for 20 min, rinsed three times with PBS, and permeabilized with 0.2% Triton X-100 for 15 min. The cells were again rinsed three times with PBS, blocked for 45 min with blocking solution (5% BSA), and then rinsed with PBS. The cells were incubated for 3 h with primary antibodies specific to mouse monoclonal anti-cytochrome c (1:100). The cells were rinsed three times in PBS and incubated in secondary antibody (FITC-conjugated goat anti-mouse secondary antibody, 1:300). The cells were washed twice in PBS and then mounted by inverting onto mounting medium on glass slides. The slides were stored at 4°C and analyzed by fluorescence microscope (Olympus).

## Electronic supplementary material

Below is the link to the electronic supplementary material.

Supplementary material 1 (PDF 122 kb)

## Abbreviations

G-Rh2: Ginsenoside Rh2
MTT: 3-(4,5-dimethylthiazol-2-yl)-2,5-diphenyltetrazoniumbromide
PARP: poly(ADP-ribose)polymerase
siRNA: small interfering RNA
