# Supplementary material for: p53-dependent Fas expression is critical for Ginsenoside Rh2 triggered caspase-8 activation in HeLa cells
Source: Protein Cell. 2014 Mar 13;5(3):224–34. doi: 10.1007/s13238-014-0027-2 (PMC3967063; doi:10.1007/s13238-014-0027-2)
Supplement: Supplementary file 1 — Supplementary material 1 (PDF 122 kb) [file 13238_2014_27_MOESM1_ESM.pdf]

Supplemental Table 1 Nucleotide sequences of the primers used for RT-PCR.

Genes	Upper: forward (5' to 3')<br>Lower: reverse (5' to 3')	Product size (bp)
Fas	CATGGCTTAGAAGTGGAAAT<br>ATTTATTGCCACTGTTTCAGG	339
FasL	ATGTTTCAGCTCTTCCACCTACAGAAGGA<br>CAGAGAGAGCTCAGATACGTTGAC	498
TNF-α	TCCTTCAGACACCCTCAACC<br>AGGCCCCAGTTTGAATTCTT	206
TNF-R1	ATGGGCCTCTCCACCGTGCCTGACC<br>GTGCCACCTCTCTGCGGGGAGCC	946
TNF-R2	ATGGCGCCCGTCGCCGTCTGG<br>GCTCCCTTCAGCTGGGGGGCTGG	762
DR4	CAGTGACTCCGAATCCCGGGAGCGC<br>GAGCATTGTCCTCAGCCCCAGGCCC	843
DR5	GTGCTCGTTGTCGCCGCGGTCCTG<br>CGGCTCCCAGGAGTCAAAGGGCAC	954
TRAIL	GAGGATCCCATGGCTATGATGGAGGTCCAG<br>GGAATTCGGCCAACTAAAAAGGCCCCGAAA	866
β-Actin	AGGGATGTGCAAGGCAGGCTTTGGTGGTGA<br>TGGTCCGTCCTGAGGCATAGAGGGACAGCA	404

Supplemental Table 2 siRNA sequences used for interference

Target genes	siRNA Duplex	
	Sense (5' to 3')	Antisense (5' to 3')
Fas	GAGAGUAUUACUAGAGCUU	AAGCUCUAGUAAUACUCUC
TNF-R1	GCUGUGGACUUUUGUACAU	AUGUACAAAAGUCCACAGC
p53	CACUACAACUACAUGUGUA	UACACAUGUAGUUGUAGUG
None	CCUACGCCACCAAUUUCGU	ACGAAAUUGGUGGCGUAGG

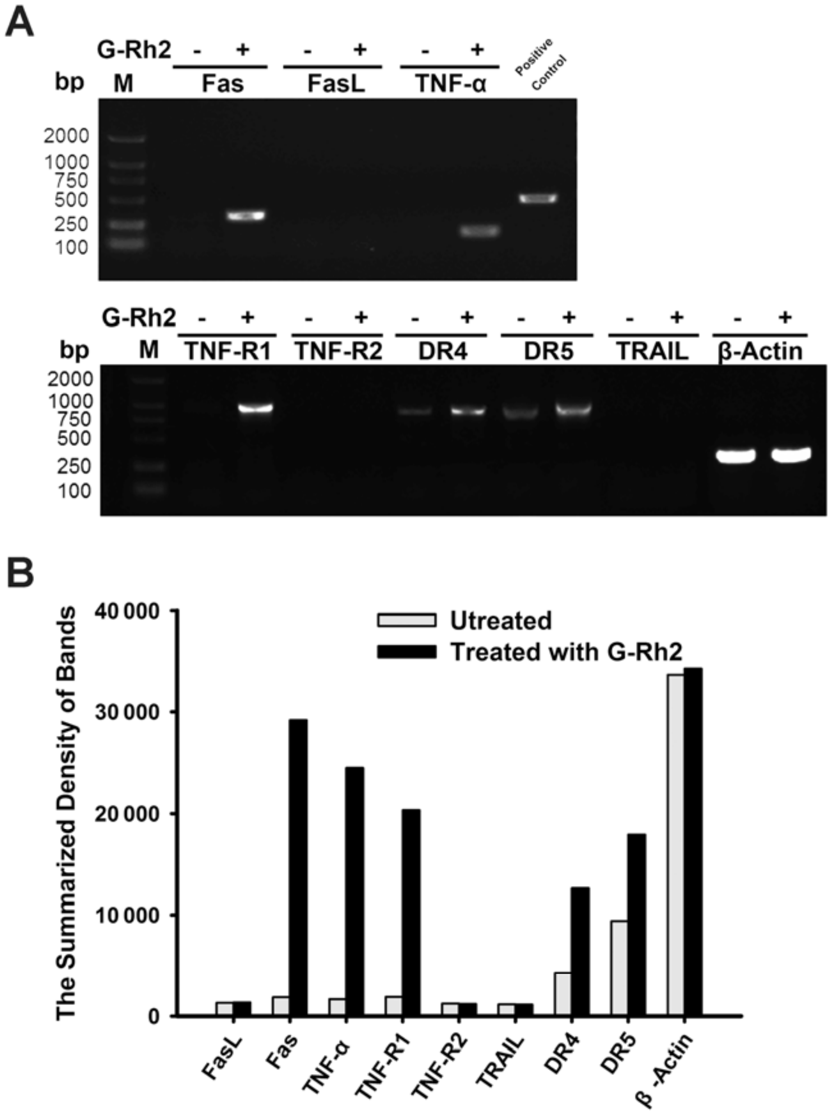

Supplemental Fig. S1 Transcriptional change of death receptors in G-Rh2-treated HeLa cells. HeLa cells were treated with or without 7.5 μg/mL G-Rh2 for 4 h. The Total RNA was extracted and analyzed by RT-PCR for the transcription of Fas, FasL, TNF-α, TNF-R1, TNF-R2, TRAIL, DR4, DR5 and β-actin. (A) The PCR products were resolved on 1.5% agarose gels and visualized with ethidium bromide under ultraviolet light. (B) Transcriptional variation was determined by the summarized density of each band using the analyzing software Image-Pro Plus.
